# α-CAs from Photosynthetic Organisms

**DOI:** 10.3390/ijms231912045

**Published:** 2022-10-10

**Authors:** Emma Langella, Anna Di Fiore, Vincenzo Alterio, Simona Maria Monti, Giuseppina De Simone, Katia D’Ambrosio

**Affiliations:** Istituto di Biostrutture e Bioimmagini, CNR, Via Pietro Castellino 111, 80131 Napoli, Italy

**Keywords:** carbonic anhydrases, metalloenzymes, photosynthetic organisms, carbon-concentrating mechanism

## Abstract

Carbonic anhydrases (CAs) are ubiquitous enzymes that catalyze the reversible carbon dioxide hydration reaction. Among the eight different CA classes existing in nature, the α-class is the largest one being present in animals, bacteria, protozoa, fungi, and photosynthetic organisms. Although many studies have been reported on these enzymes, few functional, biochemical, and structural data are currently available on α-CAs isolated from photosynthetic organisms. Here, we give an overview of the most recent literature on the topic. In higher plants, these enzymes are engaged in both supplying CO_2_ at the Rubisco and determining proton concentration in PSII membranes, while in algae and cyanobacteria they are involved in carbon-concentrating mechanism (CCM), photosynthetic reactions and in detecting or signaling changes in the CO_2_ level in the environment. Crystal structures are only available for three algal α-CAs, thus not allowing to associate specific structural features to cellular localizations or physiological roles. Therefore, further studies on α-CAs from photosynthetic organisms are strongly needed to provide insights into their structure–function relationship.

## 1. Introduction

Carbonic anhydrases (CAs) are ubiquitous enzymes that catalyze both the hydration of carbon dioxide and bicarbonate dehydration, as schematically reported in the following reaction:

$${\mathrm{CO}}_{2}+H_{2}O\;⇄{\mathrm{HCO}}_{3}^{-}+H^{+}$$

Their catalytic action allows the CO_2_ hydration reaction to speed up to 1,000,000 times per second (K_cat_ is within the range 10^4^–10^6^ s^−1^) compared to the uncatalyzed reaction [1,2,3,4]. Since the CA active site contains a metal ion, these enzymes are referred to as metalloenzymes. Most of the time the metal ion is a Zn^2+^ ion, but some CA classes also utilize Fe^2+^, Mn^2+^, Cd^2+^ and Co^2+^ ions [5].

The known CAs are grouped into eight distinct families, namely α, β, γ, δ, ζ, η, θ, and ι, which are phylogenetically unrelated and possess little to no sequence or structural similarity [6,7]. In particular, α-class is mainly present in animals and higher plants, algae, protozoa, fungi and bacteria [8,9,10]; members of β-class are found in plants, algae, cyanobacteria and also in non-photosynthetic organisms [6,9,11,12,13,14], but are absent in animals; γ-CAs have been found in archaea, some bacteria, and plants [15], whereas δ- and ζ-CAs only in diatoms and coccoliths [7,16,17,18,19,20,21]. Finally, η-, θ- and ι-CAs are the most recently discovered classes, η-CAs being found in *Plasmodium* species [22], θ-CAs in chlorophytes and diatoms [23] and ι-CAs in diatoms, algae, bacteria, and archaea [24,25].

The α-class is the most populous among the different CA families. These enzymes play a key physiologic role in all living organisms where they have been found. For example, in humans, these enzymes are involved in pH and CO_2_ homeostasis, respiration and transport of CO_2_/bicarbonate, electrolyte secretion in many tissues/organs, biosynthetic reactions, bone resorption, calcification, and tumorigenicity [26,27,28,29,30]. In protozoa, fungi, and bacteria they ensure the bicarbonate necessary for the metabolism or are involved in pH regulation [26], whereas in photosynthetic organisms such as higher plants, algae and cyanobacteria, these enzymes are mainly involved in photosynthesis by participating in photosynthetic reactions [6,8,31] and/or in the carbon-concentrating mechanism (CCM) [32,33,34,35], which is an adaptive strategy for carbon acquisition, to survive when the CO_2_ concentration limits photosynthesis [32]. Indeed, the reaction catalyzed by CAs allows to achieve a high concentration of CO_2_ near Rubisco (Ribulose Bisphosphate Carboxylase/Oxygenase), the main carboxylation enzyme in photosynthetic carbon fixation, even under limiting external inorganic carbon (Ci) [36]. Consequently, the activity of Rubisco increases, enhancing the rate of carboxylation and suppressing photorespiration events [37,38,39]. The role of cyanobacterial α-CAs as sensors to detect changes in the environment’s CO_2_ level has also been proposed [40,41,42].

α-CAs have been widely studied and a huge number of original papers and review articles have been published on these enzymes; however, most of these studies have been dedicated to human and bacterial enzymes and focused on drug design approaches [43,44]. In this review, we fill the gap existing in the α-CAs present in photosynthetic organisms, summarizing their physiological roles, cellular locations, and biochemical and structural features.

## 2. Higher Plants

Higher plants generally contain multiple gene copies of α-, β- and γ-CAs within all tissues [45]. Among these, the α-CA gene family is widely present, being detected in both cytosol and chloroplast, even though so far only a few corresponding proteins have been comprehensively characterized [46,47,48]. The largest number of studies have been carried out on *Arabidopsis thaliana*, as this plant is widely used as a model organism. Within its genome eight genes encoding α-CAs have been revealed; among these, four have been functionally investigated and one underwent a partial biochemical characterization [49]. Clear information on the intracellular location of these α-CA isoforms is available only for α-CA1, which is located in chloroplast stroma [46], and α-CA4 and α-CA5 found in thylakoid membranes [50,51,52], whereas recent studies provided evidence of the presence of α-CA2 in thylakoid membranes too [53,54,55].

Regarding their physiological function, it has been suggested that α-CA1 is involved in the transformation in chloroplast stroma of HCO_3_^−^ to CO_2_ to supply it at the active site of Rubisco [56,57]. Interestingly, this protein is one of the few plant proteins known to be targeted to the chloroplast through the secretory pathway. It contains several glycosylation sites that must be occupied by N-glycans for correct folding, trafficking, and functionality of the protein. In addition, the protein must be stabilized by a disulfide bridge between the conserved Cys27 and Cys191 residues for folding and endoplasmic reticulum (ER)-export [46,49].

As for α-CA2 and α-CA4, all the literature data obtained so far indicate that they are strongly associated with photosynthetic reactions. In particular, the comparison of different properties of *A. thaliana* wild type plants with the knockout mutants lacking α-CA2 and α-CA4 suggested that both these proteins participate, competitively, in proton exchange close to photosystem II (PSII), controlling the protonation level of the PsbS protein [53]. This protein is a key player in dissipating excess light energy *via* the regulation of non-photochemical quenching (NPQ), one of the main mechanisms ensuring that the photosynthetic apparatus is protected from photoinhibition [58]. In particular, α-CA4, catalyzing the CO_2_ hydration, under high light supplies protons to PsbS protein, determining a conformational change in the light-harvesting antenna and consequently an increase in NPQ. α-CA2 has an opposite role; indeed, this protein, catalyzing the HCO_3_^−^ dehydration, ensures fast deprotonation of PsbS under low light to avoid energy dissipation when the amount of light energy is low [53]. The presence of α-CA4 in PSII membranes and its involvement in determining proton concentration in this district have also been confirmed by more recent studies [54,59]. Moreover, it has been shown that the expression levels of the gene encoding α-CA4 increased significantly in response to the increase in light intensity [57], in agreement with the hypothesis of its role in the regulation of NPQ and in the protection of photosynthetic apparatus from photoinhibition [58].

Finally, it has been recently demonstrated that α-CA5 takes part in the photophosphorylation stimulation in the presence of bicarbonate excess in thylakoids [51,52]. Moreover, its participation in the conversion of bicarbonate to CO_2_ with the aim of supplying the latter to the membrane-bound Rubisco has been also hypothesized, thanks to its position on the stromal surface of stromal thylakoid membranes [55].

α-CAs have been found also in other plants, such as the α-CA Sb5G039000 expressed in *Sorghum bicolor* anthers [8,60], and the α-CAs Mt1g059900 and Mt1g059940 expressed in *Medicago trunculata* root nodules [8,61], but to date, an indication of their physiological function is not available.

## 3. Algae

Many α-CAs have been so far identified in photosynthetic algae and some of them have been widely characterized both from a functional and structural point of view [6,62,63]. Most of the available data concern enzymes found in green and red algae and will be described in detail in the following paragraphs. Putative α-CA encoding genes have also been identified in the genome sequences of the marine diatoms *Phaeodactylum tricornutum* and *Thalassiosira pseudonana*. However, the sequence alignment of the corresponding proteins with α-CAs from other organisms shows that none of the predicted active sites possess the three zinc-coordinating histidine residues [64,65], leaving doubts about their actual classification as α-CAs. For this reason, these enzymes will not be discussed in this review.

**Figure 1:**
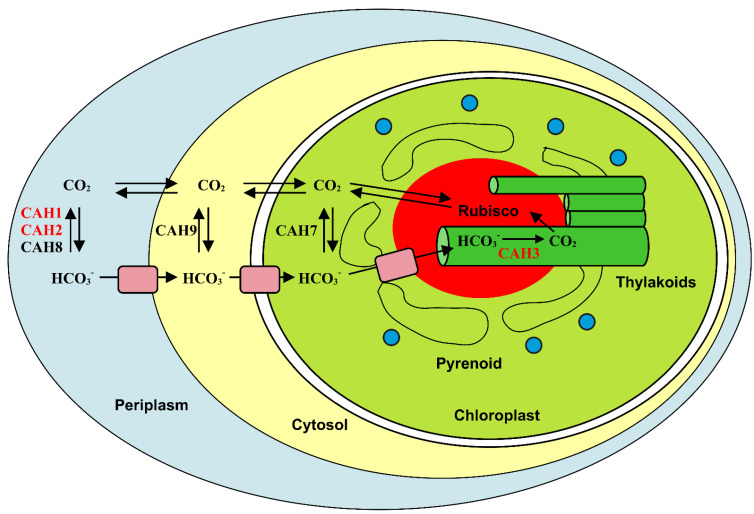
Representation of CCM in *C. reinhardtii* showing the subcellular localization of the different CAs involved. CAH1, CAH2, and CAH3 belong to the α-class and are shown in red, while CAH7, CAH8, and CAH9 are β-CAs and are colored in black. CAH7 localization is not fully demonstrated. Pink rectangles represent bicarbonate transporters whereas blue circles symbolize low CO_2_-inducible proteins (β-CAs).

*Clamidomonas reinhardtii* is a unicellular green alga that has been extensively studied in recent years, enhancing the understanding of CCM in green algae (Figure 1) [63]. This alga encodes for several CAs, belonging to α-, β- and γ-classes, which are involved in the CCM and photosynthesis. In particular, three α-CAs, namely CAH1, CAH2 and CAH3, have been identified [66,67,68,69,70,71,72]. CAH1 and CAH2 are localized in the periplasm [66,73,74], while CAH3 was found in the thylakoid lumen [75,76]. CAH1 expression is induced under low CO_2_ conditions in the presence of light, while CAH2 is poorly expressed under low CO_2_ and slightly up-regulated under high CO_2_ [31,69]. Finally, CAH3 is constitutively expressed, not showing a strong response to modifications in the CO_2_ level [62].

Studies on the inhibition of periplasmic CAs of *Chlamydomonas*, using acetazolamide (AZA) and the membrane impermeant CA inhibitor dextran bound sulphonamide (DBS), showed a decrease in cell affinity for Ci and the inhibition of the Ci-dependent O_2_ evolution [77,78]. Therefore, it was suggested that CAH1 and CAH2 are involved in CCM, facilitating the diffusion of the Ci to the plasma membrane [62,79].

CAH3 is proposed to play a critical role in CCM of *Chlamydomonas*, being responsible for the rapid conversion of HCO_3_^−^ to CO_2_ in the acidic lumen of intra-pyrenoid thylakoids, thus increasing the concentration of CO_2_ around Rubisco and consequently enhancing photosynthetic efficiency [79,80]. However, some evidence has been provided for the involvement of CAH3 in PSII activity as well [71], suggesting that under the light this enzyme, associated with the donor side of PSII, promotes the removal of protons transferred from the active site of the PSII water-oxidizing complex (WOC) to the lumen, catalyzing the bicarbonate dehydration reaction [71]. This avoids local acidification close to the WOC active site, thus improving its functioning. The recent study by Terentyev and colleagues carried out using specific CA inhibitors, namely trifluoromethanesulfonamide (TFMSA), ethoxyzolamide (EZA), and AZA, and measuring the pH-dependent change in PSII activity, provided further support to this hypothesis [81]. On the other side, Blanco-Rivero and co-workers reported that CAH3 is post-transcriptionally regulated *via* phosphorylation/dephosphorylation [47]. They also hypothesized that CAH3 remains associated with PSII in stromal thylakoids in high CO_2_ conditions, but under low CO_2_ it becomes phosphorylated and is concentrated in the intra-pyrenoid thylakoid. Thus, it seems that the function of CAH3 is related to its location within thylakoids, which in turn is regulated by post-translational modifications [32].

**Figure 2:**
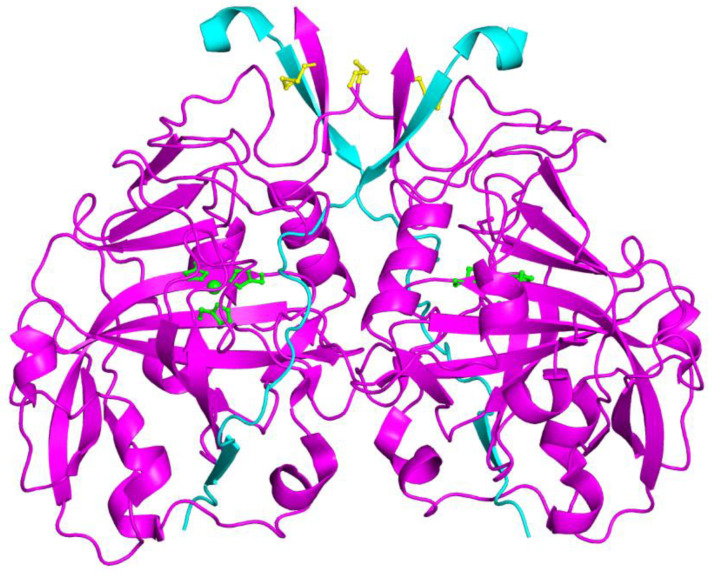
Cartoon representation of the CAH1 dimer. For both monomers, the big subunits are colored in magenta, while the small subunits in cyan. Disulfide bonds between the two monomers and between big and small subunits are reported in yellow. The zinc ions and their coordinating histidines are also shown in green.

**Figure 3:**
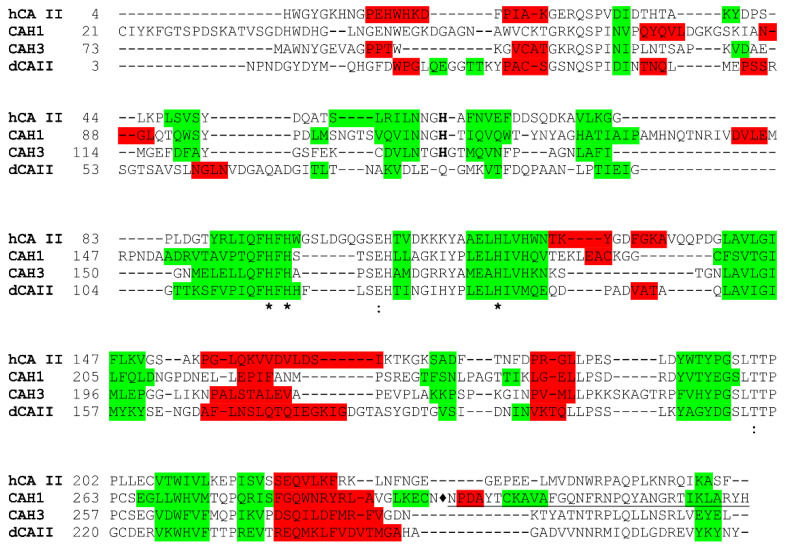
Structure-based sequence alignment of hCA II with the green algal α-CAs with known structure: CAH1 and CAH3 from *C. reinhardtii* and dCAII from *D. salina*. Histidine proton shuttles are represented in bold, while zinc coordinating histidines and gatekeeper residues Thr199 and Glu106 (hCA II numbering) are indicated with (*) and (:), respectively. For CAH1, residues of the small subunit (345–377) are underlined and ♦ indicates the break between small and big subunit (residues 21–297). The α-helix regions are colored in red and β-strand regions in green. Secondary structural elements are calculated with the DSSP program [84,85].

CAH1 and CAH3 were investigated also from a structural point of view by means of X-ray crystallography [82,83]. CAH1 is a glycosylated protein composed of a small and a large subunit which are linked by a disulfide bond [82]. The protein crystallizes as a dimer, with the two monomers linked together through an intermolecular disulfide bridge and several interactions between the loop regions (Figure 2) [82]. The formation of the dimer has also been confirmed in solution, although several data suggested that the protein can be a tetramer in some physiological conditions [82]. Each monomer displays the classical α-CA fold, with the active site region and all the key residues for the catalytic activity being very conserved (Figure 3). Some differences with respect to human CA II (hCA II) structure reside in the central β-sheet core which consists of 9 strands in CAH1 instead of 10 in hCA II. Moreover, the CAH1 structure shows some additional secondary structure elements with respect to hCA II in the surroundings of the β-sheet core. The crystallographic structure also reveals the existence of a network of hydrogen bonds between residues located in the small subunit and active site residues of the large subunit, in agreement with the experimental finding that the association of the small and big subunit is required for enzyme activity [70].

**Figure 4:**
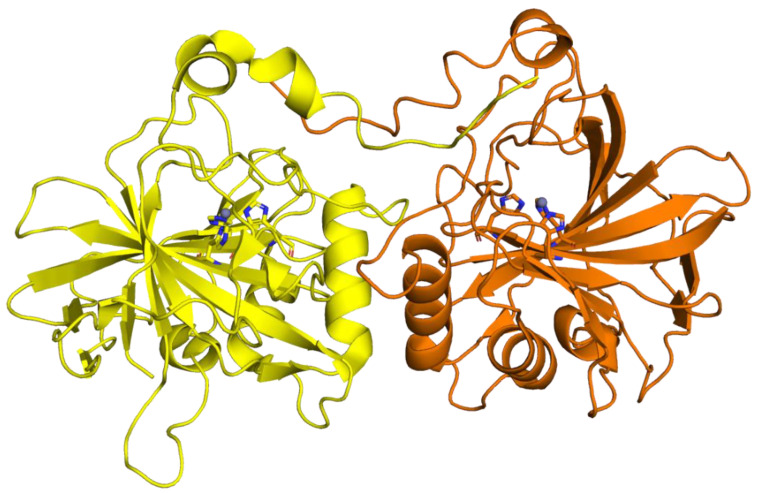
Cartoon representation of the CAH3 dimer. One subunit is colored in orange and the other one in yellow; the zinc ions and their coordinating histidines are also depicted.

CAH3 crystallizes as a dimer as well; however, in this case, the dimer is stabilized by the swap of N-terminal arms of the two monomers (Figure 4) [83]. In contrast, biochemical experiments indicated that the enzyme is a monomer in solution, even though it was suggested that in the crowded lumen under certain conditions it can occur as a dimer. CAH3 monomer retains the typical α-CA fold, and the geometry of the active site is well preserved; however, the protein surface is more hydrophobic with respect to that of human isoforms, allowing the interaction of the enzyme with the thylakoid membrane [83]. From the structural comparison with hCA II, it emerges that some sequence deletions are present in CAH3 (Figure 3). Among these, the deletion corresponding to the hCA II region 130–139 involves the rim of the catalytic cavity and is responsible for the narrowing of the active site cavity of CAH3 compared to that of the human enzyme.

**Figure 5:**
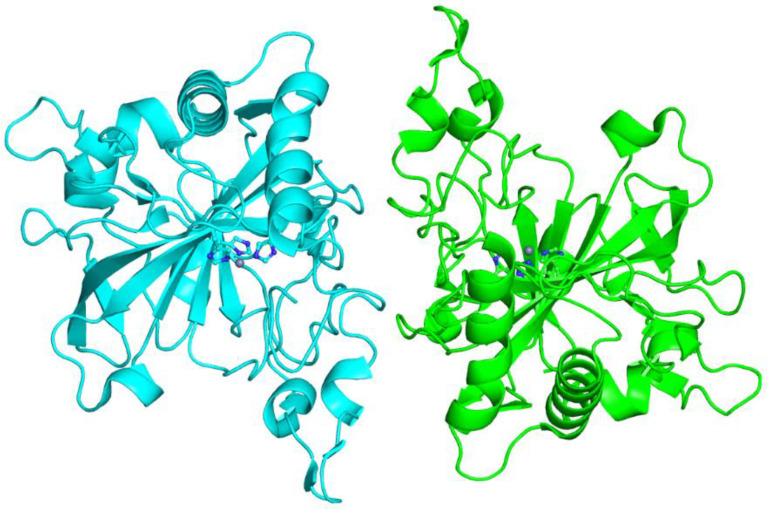
Cartoon representation of the algal dCAII dimer. One subunit is colored in cyan and the other one in green; the zinc ion and its coordinating histidines are also depicted.

*Dunaliella salina* is another unicellular green alga, capable to survive in very variable salinity conditions, from freshwater to hyper-saline lakes, such as the Dead Sea. This alga possesses two extracellular α-CAs, i.e. dCAI and dCAII, which have been proposed to be involved under limiting CO_2_ conditions in the supply of CO_2_ to the cells, obtaining it from bicarbonate [86,87]. dCAI is an unusual internally duplicated 60 kDa protein, consisting of two 52% identical α-CA domains, whereas dCAII is a single domain protein exhibiting 55% sequence identity to each dCAI domain [88]. The two proteins exhibit comparable catalytic activity and retain an active conformation over a large range of salinities [88,89]. The crystal structure of dCAII has been solved, highlighting that the protein crystallized as a dimer, with the two monomers related by a non-crystallographic two-fold axis of symmetry (Figure 5). The global fold of each monomer, characterized by a central ten-stranded β-sheet, is highly conserved although presenting some peculiar features [89]. Indeed, a comparison with other α-CAs revealed that in the dCAII structure there is a higher content of helical structure and a reduced amount of β-strand one. Moreover, the dCAII sequence shows several insertions and deletions with respect to α-CAs and the proton-shuttle His64 is missing (Figure 3) [89]. Interestingly, the solvent-accessible surface of dCAII was characterized by a reduced number of basic residues, mainly lysines, with respect to the other α-CAs, thus leading to a predominantly negative electrostatic potential surface and to a decrease in the surface hydrophobic character. It was hypothesized that the preferentially negative electrostatic potential surface could enhance protein stability and solubility in high salt concentrations. These properties are in common with the other halophilic proteins previously reported [90], although dCAII differs in its ability to maintain solubility, enzymatic activity, and correct folding even at low salt concentrations [89].

**Figure 6:**
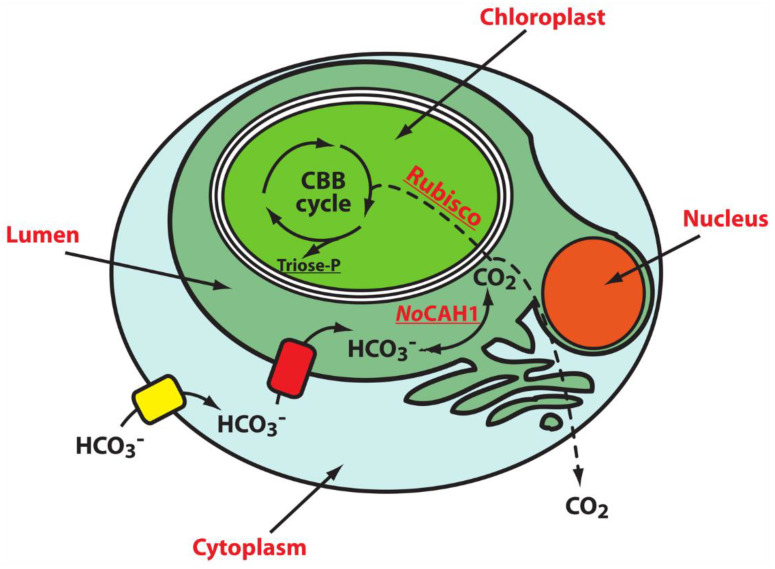
Proposed model for the *N. oceanica* CCM. The chloropast is separated from the cytoplasm by four membranes, the outermost of which, called the chloroplast ER (cER) membrane, is connected to the nuclear envelope and the ER membrane. Bicarbonate transporters push bicarbonate into the cytoplasm and then into the ER lumen, where *No*CAH1 is localized. Here, this enzyme catalyzes the formation of CO_2_, which can either diffuse into the chloroplast stroma to be fixed by Rubisco in the Calvin–Benson–Bassham (CCB) cycle, or escape from the cell (dark blue and light blue rectangles represent bicarbonate transporters).

*Nannochloropsis oceanica* is a unicellular picoplanktonic alga that represents an emerging model for research on photosynthesis and algae biology [91,92,93]. Differently from *C. reinhardtii*, which possesses the transpyrenoidal thylakoids where CAH3, one of the main players of CCM, is located (Figure 1) [64,75], *N. oceanica* lacks a pyrenoid, thus suggesting that the CCM components of this alga have a different spatial configuration. In agreement with this hypothesis, recent experimental evidence indicated *No*CAH1, an α-CA localized in the lumen of the epiplastid ER, as an essential component of the CCM in *N. oceanica* [6,36]. In particular, the proposed model assumes that bicarbonate transporters pump HCO_3_^−^ into the cytoplasm and then into the ER lumen, where *No*CAH1 accumulates. Thus, the protein catalyzes the formation of CO_2_ that either diffuses into the chloroplast stroma to be fixed by Rubisco or escapes from the cell (Figure 6) [36]. Accordingly, with this model, *No*CAH1 expression was regulated by the concentration of external Ci at both the protein and transcript levels [36].

Moreover, *Gracilariopsis chorda*, an agar-producing multicellular marine red algal species, contains four α-CAs, namely *Gc*CAHα1, *Gc*CAHα2, *Gc*CAHα3 and *Gc*CAHα4 [94]. *In silico* analysis of these proteins using different prediction tools revealed in all of them an N-terminal sequence with a high degree of hydrophobicity that could function as a leader sequence for their targeting to the ER. In agreement with this hypothesis, when heterologously expressed in protoplasts of *A. thaliana* leaf cells, the four *Gc*CAHαs were localized in the ER and two of them (i.e. *Gc*CAHα2 and *Gc*CAHα4) were further targeted to the vacuole [94]. However, it cannot be excluded that in *G. chorda Gc*CAHα1 and *Gc*CAHα3 are initially targeted to the ER to be subsequently transported to other organelles, such as the chloroplast, and that this does not happen in *Arabidopsis* due to the difference in the mechanisms of trafficking from the endomembrane compartments to chloroplasts between red algae and *Arabidopsis* [94]. As it concerns the physiological roles of the *G. chorda* α-CAs, even if their involvement in the red alga CCM could be hypothesized, further studies are absolutely needed to clarify this point.

## 4. Cyanobacteria

**Figure 7:**
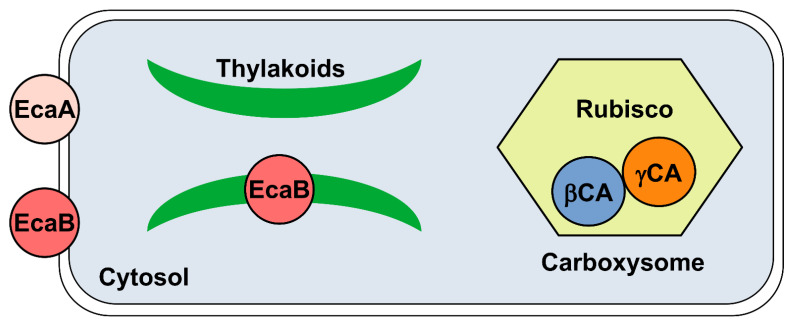
Schematic picture of cyanobacterium cell, highlighting the presence of the carboxysome and thylakoid membranes within the cytosol. Subcellular localization of CAs is also shown: EcaA (α-CA) is extracellular, EcaB (β-CA) is primarily located in the thylakoid membrane with a smaller component associated with the plasma membrane, and β- and γ-CAs are localized in the carboxysome.

Cyanobacteria are a very large group of photosynthetic bacteria, diffused in various habitats [95]. They appeared on the earth at least 3.5 billion years ago [96] and during their evolution they have been subjected to profound mutations due to deep changes in the gaseous composition of the earth’s environment, initially containing a high CO_2_ content and low O_2_ levels [33,35]. The conversion of the early oxygen-poor reducing atmosphere into an oxidizing one, with a drastic reduction in CO_2_ concentration, pushed cyanobacteria to develop alternative mechanisms for efficiently acquiring Ci for photosynthesis. In particular, they developed a very efficient photosynthetic CCM, which allowed them to survive at low CO_2_ concentrations [35,42]. The carboxysome is an essential part of the cyanobacterial CCM, unlike algae in which the pyrenoid is instead present (Figure 7) [78,97]. Cyanobacteria CAs play a central role in this mechanism [42] and up to now, α-, β- and γ-CAs [42,98] have been identified in these organisms. β- and γ-CAs are predominant, while α-CAs are less widespread [99].

**Figure 8:**
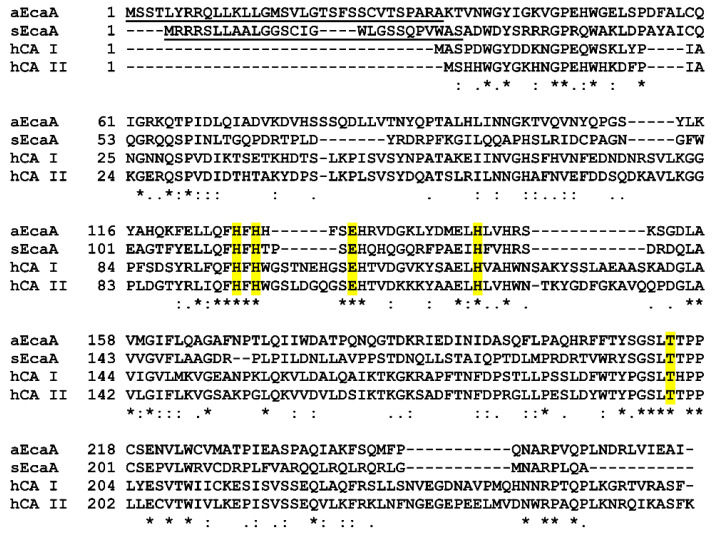
Multiple sequence alignment of *Anabaena* EcaA (aEcaA), *Synechococcus* EcaA (sEcaA), hCA I and hCA II. Conserved residues are indicated with an asterisk (*), while (:) and (.) indicate conservative and semi-conservative substitutions, respectively. Histidine residues involved in zinc coordination, Thr199 and Glu106 (hCA II numbering) are highlighted in yellow. Tat-signal peptides are underlined.

A gene encoding an α-CA (EcaA, external carbonic anhydrase alpha class) was initially isolated from the cyanobacteria *Anabaena* sp. strain PCC7120 and *Synechococcus elongatus* PCC7942 [41]. *Anabaena* EcaA is a protein of approximately 29 kDa which shows a significant amino acid sequence homology with several human α-CAs, such as hCA I and hCA II, including conservation of most active site residues required for Zn^2+^ binding and catalytic activity (Figure 8) [41,98]. A twin-arginine translocation (Tat) signal peptide is present in the N-terminal region of this protein and is responsible for protein transport to the outside of the cell [98,100]. Indeed, immunogold localization studies with polyclonal antisera directed against *Anabaena* EcaA showed that this CA has an extracellular location and is associated with the cell wall, periplasmic space or cytoplasmic membrane [41]. Expression of *Anabaena* EcaA is regulated by CO_2_ concentration in the growth medium, being highest in cells grown at elevated CO_2_ levels (1% CO_2_ in air), whereas cells transferred in medium with low levels of CO_2_ (0.01%) undergo a gradual decline in protein expression [41].

*Synechococcus* EcaA is a 26 KDa protein with high sequence homology with both human α-CAs and *Anabaena* EcaA (Figure 8), and as for the latter, it contains at the N-terminus many positively charged residues indicative of the presence of the signal peptide for membrane targeting [41]. Moreover, protein expression in this case is also influenced by CO_2_ concentration, with high levels of the enzyme occurring when cells are grown at elevated concentrations of CO_2_ [41].

Despite EcaA proteins being discovered more than twenty years ago, their CA activity was not confirmed for a long time. In 2009, Wang and coworkers [101] reported a relatively high activity of the *Anabaena* EcaA protein heterologously expressed in *E. coli*, whereas only recently clear evidence of the *in vitro* CA activity of *Synechococcus* enzyme was obtained [102]. Interestingly, differently from *Anabaena* EcaA, the *Synechococcus* EcaA contains a disulphide bond that is important for the enzyme activity, as already reported for other CAs belonging to the α-class. Indeed, the enzyme shows high specific CA activity only when it is expressed in heterologous bacterial systems which support the formation of disulfide bonds, independently from the presence or absence of the leader peptide at the N-terminus. Accordingly, the enzyme activity of recombinant *Synechococcus* EcaA expressed in bacterial systems that do not support optimal disulfide bond formation can be restored by the addition of a thiol-oxidizing agent [102].

The functional role of the EcaA proteins in cyanobacterial photosynthesis was also investigated by generating a deletion mutant of the *Synechococcus* protein. Surprisingly, both the CA activity, measured at the cell surface, and the growth rate of the mutant were comparable to those of the WT cells [41,103], suggesting that EcaA does not have a significant role in the CCM. Thus, due to its capability to bind CO_2_ and HCO_3_^−^ and to its cell-surface location, it was hypothesized that this protein could play a role as a sensor, detecting or signaling changes in the level of CO_2_ in the environment [40,41,42,98]. Additional studies are necessary to clarify this point.

Further, EcaA enzymes have been found in other cyanobacteria, such as the alkalophilic cyanobacteria *Rhabdoderma lineare* and *Microcoleus chthonoplates*, and the marine cyanobacterium *Cyanothece* sp. ATCC51142 [104,105,106]. *Cyanothece* EcaA is a highly active CA, whose enzymatic activity was confirmed both *in vivo* and *in vitro*, using intact cells and a recombinant protein expressed in a heterologous host, respectively [106]. Interestingly, even if *Cyanothece* EcaA contains two cysteine residues (Cys55 and Cys209), unlike *Synechococcus* EcaA, its enzymatic activity does not show a redox regulation, indicating the absence of the disulfide bond [102]. Inhibition studies using specific α-CA inhibitors with different membrane permeability suggested that *Cyanothece* EcaA is not associated with the outer membrane, since the rate of CO_2_ hydration was highly reduced by EZA, which is characterized by high permeability through cell membranes but not by AZA, which cannot penetrate cells [106]. Further studies, carried out in *E. coli* cells with several recombinant forms of the *Cyanothece* EcaA protein (with and without the leader peptide), confirmed the transport of recombinant proteins containing the leader peptide across the inner cytoplasmic membrane in *E. coli* cells, as also directly confirmed by immunofluorescent microscopy [107].

Finally, EcaAs isolated from *R. lineare* and *M. chthonoplates* are two extracellular α-CAs, localized in the glycocalyx membrane [105,108], probably involved in cell survival under extreme conditions of soda lakes. Indeed, it has been suggested that these enzymes may preserve the intracellular Ci pool for photoautotrophic assimilation, preventing CO_2_ leakage from the cell through its conversion into bicarbonate [99,109].

## 5. Conclusions and Future Perspectives

In this review, we have provided a comprehensive overview of experimental data existing on α-CAs present in photosynthetic organisms, highlighting that these enzymes can have different cellular localization and adopt many physiological roles. In higher plants, these enzymes have been little studied; however, available data indicate their involvement in both supplying CO_2_ at the active site of Rubisco and determining proton concentration in PSII membranes. On the contrary, a greater number of studies are available on the algal and cyanobacterial α-CAs, which have been demonstrated to be actively involved in CCM, photosynthetic reactions and detecting or signaling changes in the environment’s CO_2_ level. Even the biochemical and structural studies are limited in number and do not allow us to rationalize the presence of structural features associated with particular cellular localizations or physiological functions. From this scenario, it emerges that many studies are still needed on α-CAs from photosynthetic organisms in order to provide a clear structure–function relationship and to understand their correlation with the physiological functions exercised by the other classes of CAs present in the same organisms.

## Data Availability

Not applicable.
